# One-Step Chromatographic Purification of *Helicobacter pylori* Neutrophil-Activating Protein Expressed in *Bacillus subtilis*

**DOI:** 10.1371/journal.pone.0060786

**Published:** 2013-04-08

**Authors:** Kuo-Shun Shih, Chih-Chang Lin, Hsiao-Fang Hung, Yu-Chi Yang, Chung-An Wang, Kee-Ching Jeng, Hua-Wen Fu

**Affiliations:** 1 Institute of Molecular and Cellular Biology, National Tsing Hua University, Hsinchu, Taiwan, Republic of China; 2 Department of Medical Technology, Jen-Teh Junior College of Medicine, Nursing and Management, Miaoli, Taiwan, Republic of China; 3 Departments of Research, Taichung Veterans General Hospital, Taiwan, Republic of China; 4 Department of Life Science, National Tsing Hua University, Hsinchu, Taiwan, Republic of China; New York University, United States of America

## Abstract

*Helicobacter pylori* neutrophil-activating protein (HP-NAP), a major virulence factor of *Helicobacter pylori* (*H. pylori*), is capable of activating human neutrophils to produce reactive oxygen species (ROS) and secrete inammatory mediators. HP-NAP is a vaccine candidate, a possible drug target, and a potential *in vitro* diagnostic marker for *H. pylori* infection. HP-NAP has also been shown to be a novel therapeutic agent for the treatment of allergic asthma and bladder cancer. Hence, an efficient way to obtain pure HP-NAP needs to be developed. In this study, one-step anion-exchange chromatography in negative mode was applied to purify the recombinant HP-NAP expressed in *Bacillus subtilis* (*B. subtilis*). This purification technique was based on the binding of host cell proteins and/or impurities other than HP-NAP to DEAE Sephadex resins. At pH 8.0, almost no other proteins except HP-NAP passed through the DEAE Sephadex column. More than 60% of the total HP-NAP with purity higher than 91% was recovered in the flow-through fraction from this single-step DEAE Sephadex chromatography. The purified recombinant HP-NAP was further demonstrated to be a multimeric protein with a secondary structure of α-helix and capable of activating human neutrophils to stimulate ROS production. Thus, this one-step negative chromatography using DEAE Sephadex resin can efficiently yield functional HP-NAP from *B. subtilis* in its native form with high purity. HP-NAP purified by this method could be further utilized for the development of new drugs, vaccines, and diagnostics for *H. pylori* infection.

## Introduction

*Helicobacter pylori* (*H. pylori*), a microaerophilic Gram-negative bacterium, infects about half of the entire human population [1], [2]. Infection of *H. pylori* is associated with chronic gastritis, gastric ulcer, and gastric cancer. In *H. pylori*-infected patients with chronic gastritis, a strong infiltration of neutrophils was detected in their gastric mucosa, and the extent of gastric neutrophil infiltration was correlated to the degree of mucosa damage [3], [4]. *H. pylori* neutrophil-activating protein (HP-NAP) is a virulence factor that recruits neutrophils to inflamed mucosal tissue during *H. pylori* infection. It was first characterized by its ability to promote neutrophil adherence to endothelial cells and induce the production of reactive oxygen species (ROS) by neutrophils [5]. In addition to acting as a chemoattractant to induce neutrophil migration, HP-NAP can cross the endothelium to promote neutrophil-endothelial cell adhesion [6], [7]. Thus, HP-NAP could play a critical role in recruiting neutrophils towards the infected area to trigger the gastric inflammatory response during *H. pylori* infection.

HP-NAP is a 150 kDa oligomer identified from the water extract of *H. pylori* [5]. Crystal structure analysis shows that HP-NAP is a spherical dodecameric protein consisting of twelve identical monomers with a central iron-binding cavity [8]. Each monomer is a 17 kDa protein with a four-helix bundle structure [8], [9]. The surface of HP-NAP is characterized by the presence of a large number of positively charged residues [8], which might be important for neutrophil activation. HP-NAP not only triggers the production of ROS but also induces the secretion of chemokines and cytokines from neutrophils and monocytes. HP-NAP induces the synthesis and release of CXCL8 (interleukin-8, IL-8), CCL3 (MIP-1α), and CCL4 (MIP-1β) by neutrophils [7] and the production of tissue factor, tumor necrosis factor alpha (TNF-α), interleukin-6 (IL-6), and IL-8 by monocytes [10], [11]. HP-NAP also contributes to T helper 1 (Th1) polarization by stimulating the production of IL-12 and IL-23 by neutrophils and monocytes [11]. These various inflammatory responses induced by HP-NAP support the idea that HP-NAP plays an important role in immunity and pathogenesis.

HP-NAP has been reported to be a protective antigen of *H. pylori* [6]. The recombinant HP-NAP was used as one of the components of a protein vaccine with therapeutic effect against *H. pylori* infection in the beagle dog model [12]. This protein vaccine has been demonstrated to be safe and immunogenic in humans and may be used for immunoprophylaxis against *H. pylori* infection [13]. In addition to vaccine development, recombinant HP-NAP could be applied in the treatment of allergic diseases and immunotherapy of cancer due to its ability to induce Th1 responses. HP-NAP has been used as an immune modulating agent to suppress Th2 responses in ovalbumin-induced allergic asthma and *Trichinella spiralis* infection [14], [15] and to inhibit the growth of bladder cancer [16]. Moreover, HP-NAP is a potential target used for development of an enzyme-linked immunosorbent assay (ELISA) for clinical diagnosis through detection of the antibodies against HP-NAP in humans. Such an HP-NAP-based ELISA has already been applied either to detect serum antibodies against HP-NAP in *H. pylori*-infected patients [17] or to study the association of anti-HP-NAP antibody response with gastric cancer [18] and with anti-aquaporin-4 autoimmunity related neural damage [19]. A recent finding that arabinogalactan protein extracted from Chios mastic gum (CMG) was able to inhibit HP-NAP-induced neutrophil activation further suggests that HP-NAP can act as a target for new drugs against *H. pylori* inflammation [20]. Since purified HP-NAP is required for the above mentioned applications, a strategy for efficient purification of HP-NAP with high purity needs to be developed.

HP-NAP was first purified from the water extract of *H. pylori* by two gel-filtration and one ion-exchange chromatographic steps [5]. Several studies show that recombinant HP-NAP expressed in *Escherichia coli* was purified in its native form by at least two chromatographic steps: two consecutive gel-filtration chromatography or ion-exchange chromatography followed by gel-filtration chromatography [21], [22], [23]. In this study, a method using one-step DEAE anion-exchange chromatography in negative mode has been developed for purification of HP-NAP from *B. subtillis*. Further, the physical characteristics of the purified HP-NAP and its ability to induce ROS production in neutrophils have been investigated. Our results show that this one-step chromatographic purification is an efficient method to obtain recombinant HP-NAP expressed from *B. subtillis* with high purity and biological activity.

## Results

### Expression of Recombinant HP-NAP in *B. subtilis*

**Figure 1:**
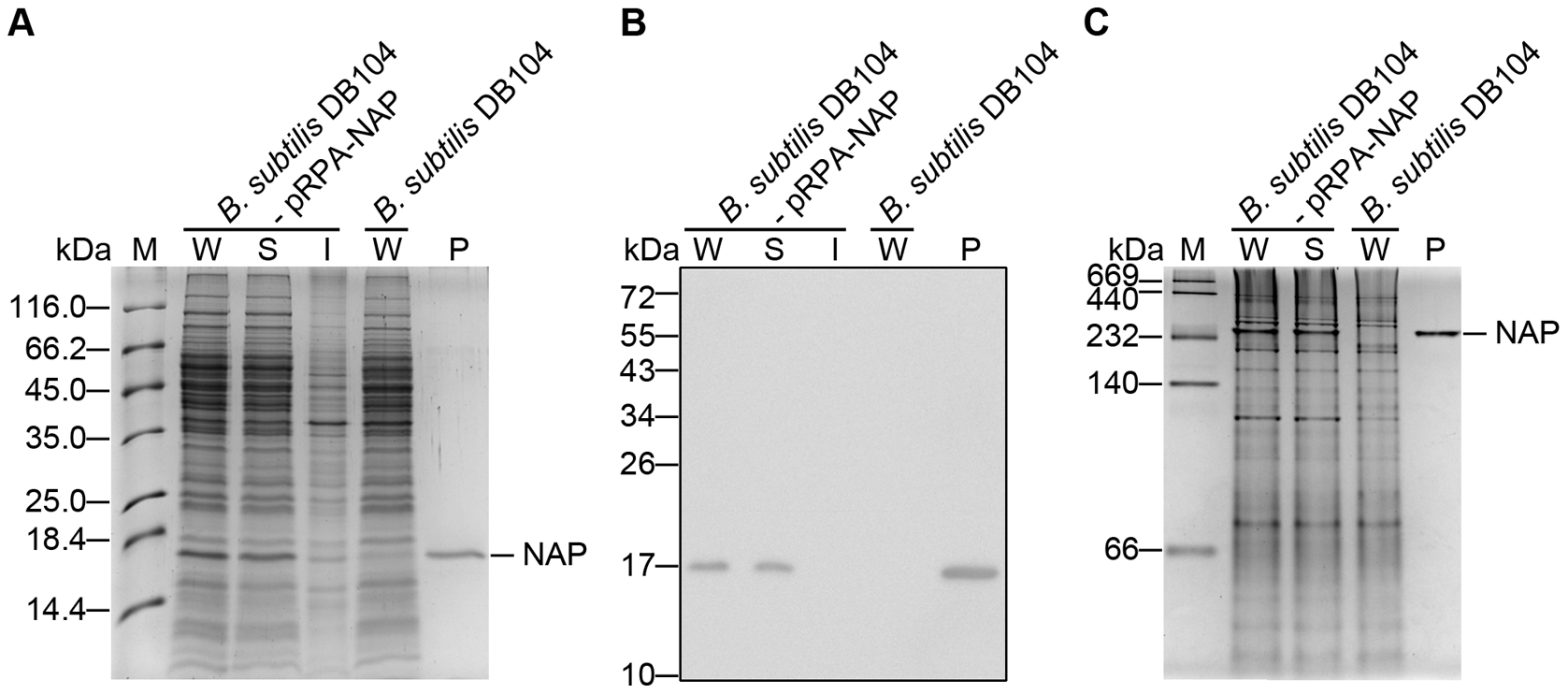
Recombinant HP-NAP expressed in *B. subtilis* as a soluble, multimeric protein. *B. subtilis* DB104-pRPA-NAP and *B. subtilis* DB104 were incubated at 37°C for 15 hr. The cells were harvested, and the whole cell lysates were prepared as described in Materials and Methods. The whole cell lysate (W) from *B. subtilis* DB104-pRPA-NAP was further centrifuged to separate the soluble fraction (S) and insoluble pellet (I). The samples were analyzed by SDS-PAGE (**A**), immunoblotting (**B**) and native-PAGE (**C**). Recombinant HP-NAP purified from *E. coli* BL21(DE3) harboring pET42a-NAP was used as a positive control (P). Molecular weights (M) in kDa are indicated on the left of the stained gels and the blot.

To avoid lipopolysaccharide contamination, a *Bacillus subtilis* DB104 expression system was employed to express *Helicobacter pylori* neutrophil-activating protein (HP-NAP). The *nap* gene was cloned into a constitutive pRPA expression vector and then transformed into *Bacillus subtilis* DB104 for protein expression. SDS-PAGE analysis showed that a marked increase in the expression of a protein with a molecular weight of approximately 17 kDa in *B. subtilis* harboring pRPA-NAP as compared to *B. subtilis* DB104 (Fig. 1A). This 17 kDa protein was confirmed to be HP-NAP and only expressed in *B. subtilis* harboring pRPA-NAP by immunoblot analysis using an anti-HP-NAP antibody, MAb 16F4 (Fig. 1B). Furthermore, the recombinant HP-NAP was present in the soluble fraction of the *B. subtilis* lysate (Fig. 1A and B). We next examined the oligomeric state of the recombinant HP-NAP expressed in *B. subtilis* by native-PAGE. As shown in figure 1C, a protein with apparent molecular weight of a little bit over 232 kDa corresponding to recombinant HP-NAP was present in the soluble fraction of the cell lysate from *B. subtilis* harboring pRPA-NAP but not from *B. subtilis* DB104. Thus, the recombinant HP-NAP was expressed as a soluble multimeric protein in *B. subtilis*.

### Purification of Recombinant HP-NAP Expressed in *B. subtilis* by One-step DEAE Anion-exchange Chromatography

**Figure 2:**
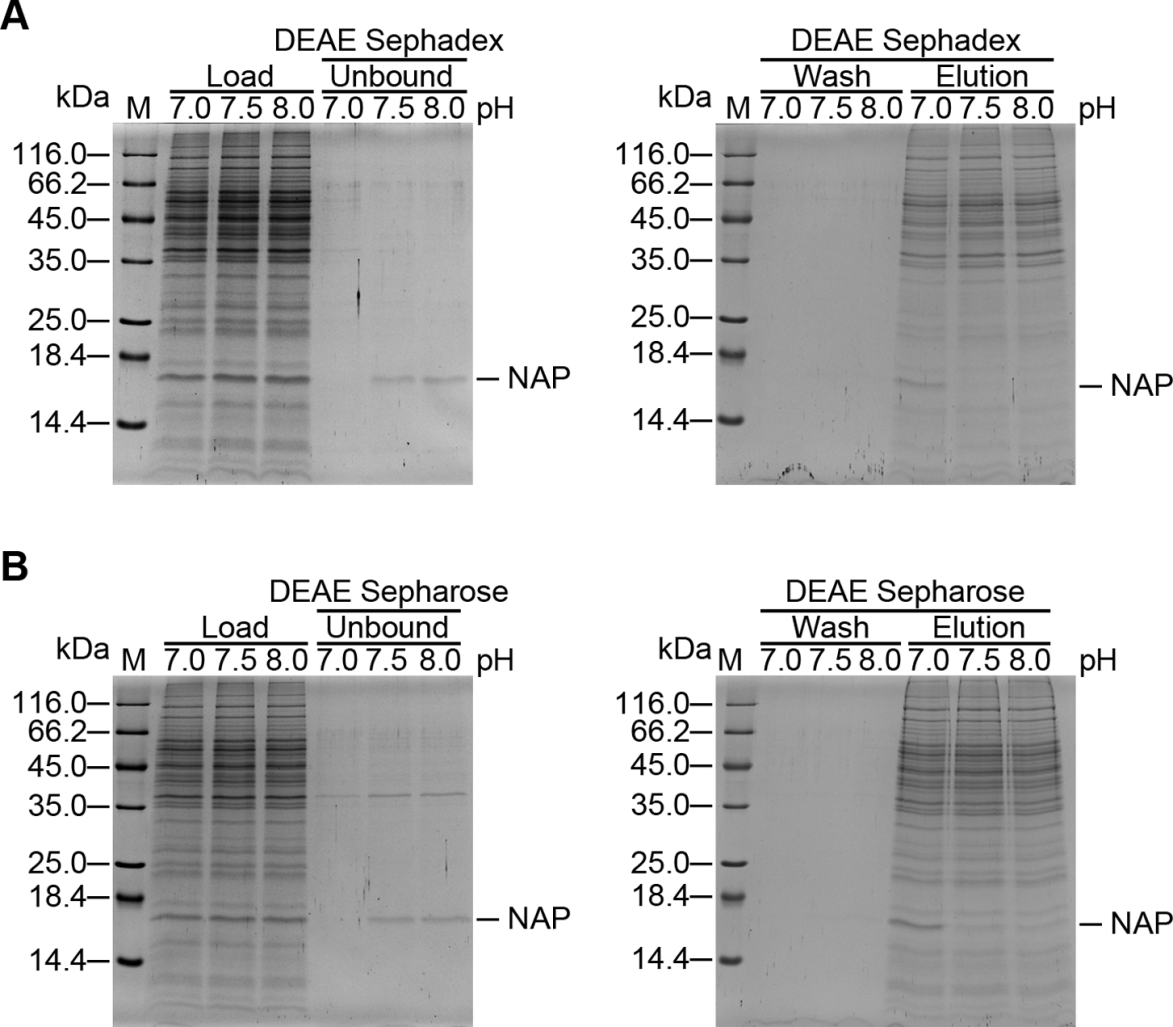
Optimization of pH values and DEAE resins for purification of recombinant HP-NAP expressed in *B. subtilis*. The soluble fraction from the whole cell lysate of *B. subtilis* DB104-pRPA-NAP was adjusted to the indicated pH values to purify recombinant HP-NAP using DEAE Sephadex and DEAE Sepharose resins by a batch method as described in Materials and Methods. The soluble fraction from the whole cell lysate of *B. subtilis* DB104-pRPA-NAP, indicated as load, and the unbound supernatant, wash fraction, and elution fraction collected using DEAE Sephadex (**A**) and DEAE Sepharose (**B**) resins were analyzed by SDS-PAGE. Molecular weights (M) in kDa are indicated on the left of the stained gels.

Due to the large molecular size of HP-NAP, gel-filtration chromatography has been applied to purify either endogenous HP-NAP from *H. pylori* [5] or its recombinant protein expressed in *E. coli* [21], [22], [23]. However, this chromatographic technique is laborious and time-consuming. Also, more than one chromatographic step is needed to purify HP-NAP with high purity. In the cell lysate of *B. subtilis*, several protein bands with molecular weights between 140 and 232 kDa were detected in the soluble fraction of *B. subtilis* expressing HP-NAP by native-PAGE analysis (Fig. 1C). It might be difficult to separate the recombinant HP-NAP from these proteins by using gel-filtration chromatography. Since the isoelectric point (pI) of HP-NAP was reported to be 6.75 [9], we chose DEAE anion-exchange chromatography to purify recombinant HP-NAP expressed in *B. subtilis* using a buffer with pH value higher than 6.75. To optimize the purification condition, we tested three different buffer pH values, 7.0, 7.5 and 8.0, in combination with either DEAE Sephadex or DEAE Sepharose resins for their feasibility to purify recombinant HP-NAP in *B. subtilis* by using a small-scale batch method. At pH 7.5 and 8.0, recombinant HP-NAP was mainly present in the unbound supernatants and reached a high degree of purity for both resins (Fig. 2). At pH 7.0, the majority of recombinant HP-NAP was detected in the bound fractions for both resins (Fig. 2). However, most of the endogenous proteins from *B. subtilis* were present in the bound fractions for both resins regardless of the pH values investigated (Fig. 2). At the three pH values tested, recombinant HP-NAP kept its multimeric structure (Fig. S1). This finding raises the possibility that recombinant HP-NAP could be purified from the unbound fraction rather than the bound fraction, as traditionally applied for ion-exchange chromatography. When the purities of recombinant HP-NAP in the unbound fractions of the two resins were compared, DEAE Sephadex resin showed better performance than DEAE Sepharose resin in purification of recombinant HP-NAP at both pH 7.5 and 8.0 (Fig. 2). Thus, negative chromatography could be applied to isolate highly pure recombinant HP-NAP from *B. subtilis* through the collection of the unbound fractions using DEAE Sephadex resin at pH 7.5 and 8.0.

**Figure 3:**
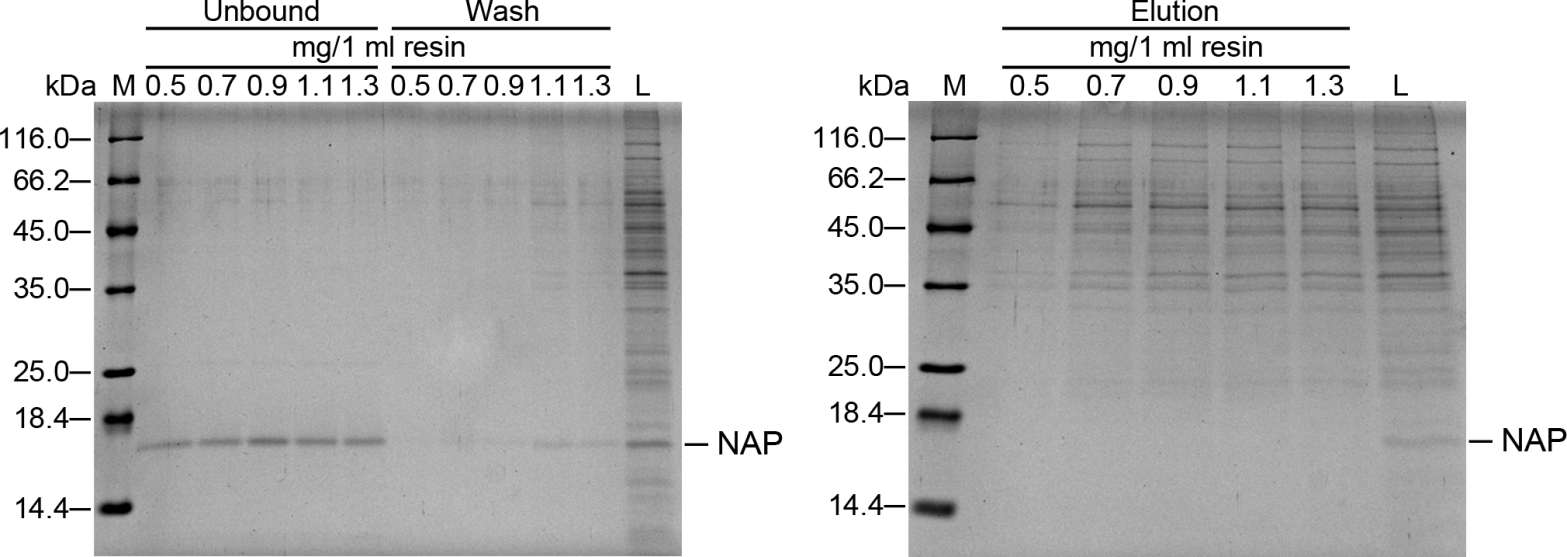
Optimization of the ratio of the amount of proteins loaded onto DEAE Sephadex resins for purification of recombinant HP-NAP expressed in *B. subtilis*. The soluble fraction from the whole cell lysate of *B. subtilis* DB104-pRPA-NAP was adjusted to pH 8.0. This sample, indicated as load (L), was then loaded onto DEAE Sephadex resins according to the indicated ratio of mg proteins per milliliter of resins to purify recombinant HP-NAP by a batch method as described in Materials and Methods. The soluble fraction from the whole cell lysate of *B. subtilis* DB104-pRPA-NAP, the unbound supernatant, wash fraction, and elution fraction were analyzed by SDS-PAGE. Molecular weights (M) in kDa are indicated on the stained gels.

To develop a strategy by using negative chromatography to purify recombinant HP-NAP expressed in *B. subtilis* in one step, we optimized the amount of proteins from the whole cell lysate of *B. subtilis* DB104-pRPA-NAP loaded onto DEAE Sephadex resins at pH 8.0 by a small-scale batch method to obtain a maximum yield of pure recombinant HP-NAP. At the loading ratios ranging from 0.5 to 1.3 mg of proteins per milliliter of resins, the majority of recombinant HP-NAP remained in the unbound supernatants (Fig. 3). However, the endogenous proteins from *B. subtilis* were gradually increased in the wash fractions when the ratio of protein to resin increased from 0.9 to 1.3 mg/ml (Fig. 3). Even though most of the endogenous proteins from *B. subtilis* were present in the bound fractions, the binding of these proteins to the resins had achieved the maximum level with the ratio of protein to resin reaching 0.7 mg/ml (Fig. 3). These results indicated that the optimal loading ratio of the amount of proteins from the whole cell lysate to the volume of DEAE Sephadex resins should be below 0.7 mg/ml at pH 8.0.

**Figure 4:**
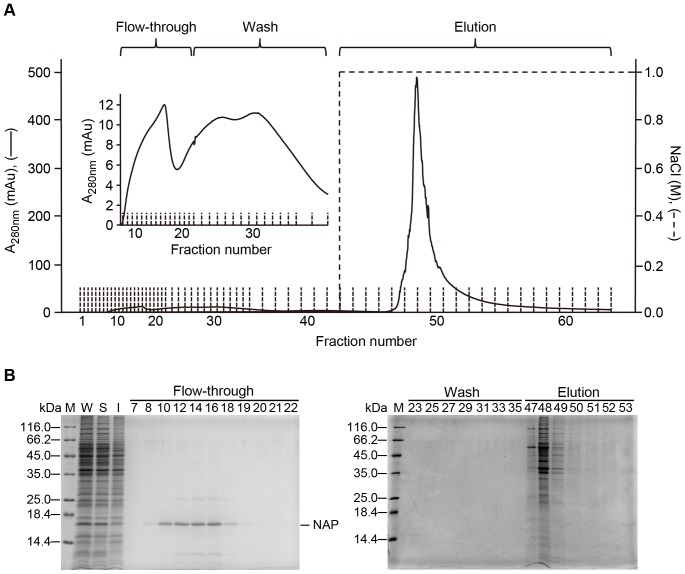
Purification of recombinant HP-NAP from *B. subtilis* by DEAE Sephadex anion-exchange chromatography. A, The soluble fraction from the whole cell lysate of *B. subtilis* DB104-pRPA-NAP was applied to DEAE Sephadex anion-exchange column as described in Materials and Methods. The chromatogram was recorded by UV absorbance at 280 nm. The fractions of flow-through, wash, and elution were ranged from fractions 1 to 22, 23 to 42, and 43 to 63, respectively. The inset represents the enlarged chromatogram of fractions 1 to 37. B, The whole cell lysate (W), soluble fraction (S), and insoluble pellet (I) of *B. subtilis* DB104-pRPA-NAP and the selected fractions corresponding to the fraction number of chromatogram shown in (**A**) were analyzed by SDS-PAGE. Molecular weights (M) in kDa are indicated on the left of the stained gels.

**Figure 5:**
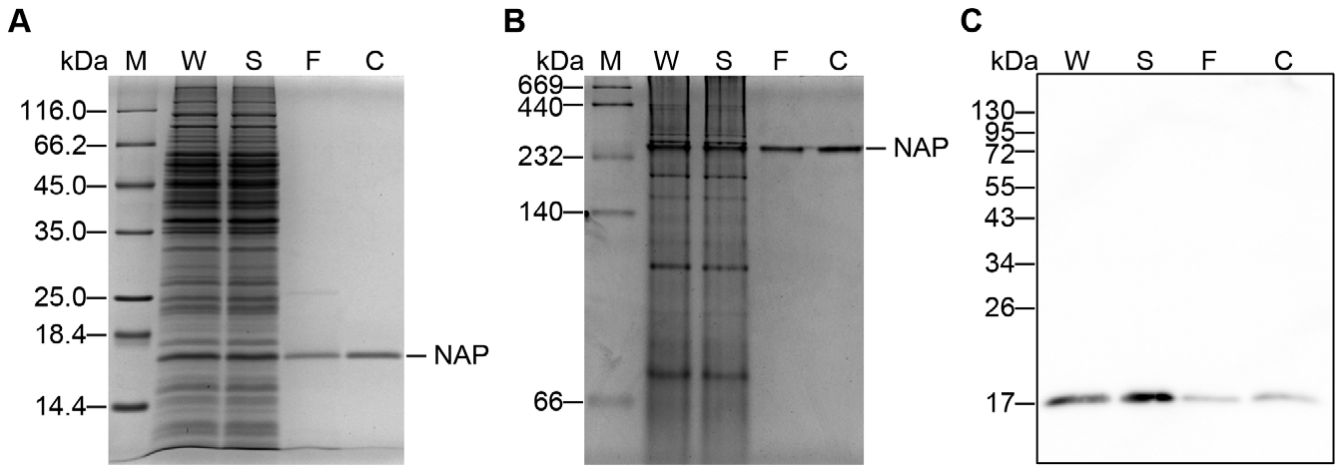
PAGE and immunoblot analysis of the purification process of recombinant HP-NAP expressed in *B. subtilis*. The whole cell lysate (W) and soluble fraction (S) of *B. subtilis* DB104-pRPA-NAP, HP-NAP-containing flow-through fractions (F) obtained by DEAE Sephadex chromatography, and the concentrated HP-NAP (C) from flow-through fractions were analyzed by SDS-PAGE (**A**), native-PAGE (**B**), and immunoblotting (**C**) with an anti-HP-NAP antibody (MAb 16F4). Molecular weights (M) in kDa are indicated on the left of stained gels and the blot.

We then applied this negative chromatography approach to purify the recombinant HP-NAP expressed in *B. subtilis* using a DEAE Sephadex column at pH 8.0 by keeping the ratio of protein to resin below 0.7 mg/ml. As expected, the majority of recombinant HP-NAP was present in the flow-through fractions (Fig. 4). Most of the endogenous proteins from *B. subtilis* were eluted from DEAE Sephadex resins under the high salt condition (Fig. 4). After the HP-NAP-containing flow-through fractions were concentrated, coupled with a simultaneous buffer exchange using an ultrafiltration membrane with a higher-molecular-weight cutoff, the purity of recombinant HP-NAP was further increased (Fig. 5A). The purified HP-NAP was expressed as a multimeric protein with molecular weight of ∼232 kDa by native-PAGE analysis (Fig. 5B). Immunoblotting analysis further showed that HP-NAP was remained in the flow-through fractions and enriched after a final concentration step (Fig. 5C). The details of a typical purification of recombinant HP-NAP from *B. subtilis* are summarized in Table 1. The purity of recombinant HP-NAP was greatly elevated to 94.3% after the step of ion-exchange chromatography using DEAE Sephadex resin, by which a 16-fold purification with a 63% recovery was achieved, supporting the fact that this negative chromatography approach was an efficient step in the purification procedure. Concentration and buffer exchange did not affect the yield of recombinant HP-NAP, whereas removal of endotoxin reduced the overall yield by ∼5.8% (Table 1). The final yield of the pure recombinant HP-NAP ranged from 1.3 to 1.5 mg per gram of *B. subtilis* cell paste. The overall purity of HP-NAP was >92% in the preparations using this purification procedure. Thus, recombinant HP-NAP can be efficiently purified from *B. subtilis* by one-step negative chromatography using DEAE Sephadex anion-exchange resin.

**Table 1:** Purification summary table of recombinant HP-NAP from *B. subtilis.*

Purification step	Total protein (mg)^b^	Volume (mL)	Purity (%)^c^	Amount of HP-NAP (mg)^d^	Recovery of HP-NAP (%)^e^
**Whole cell lysate** a	45.09	27.00	4.94	2.23	100.00
**Supernatant**	38.07	27.00	5.64	2.15	96.41
**DEAE Sephadex chromatography**	1.44	36.00	94.33	1.36	60.99
**Stirred ultrafiltration**	1.44	4.80	95.38	1.37	61.43
**Acrodisc syringe filtration**	1.30	4.65	95.37	1.24	55.61

a From 0.84 g of *B. subtilis* cell paste obtained from 300 ml of bacterial culture.

b Protein concentration determined by Bradford method with bovine serum albumin as the reference.

c Values determined from densitometry measurement as described in Materials and Methods.

d Values determined by multiplying the values in the columns of “otal protein” and “Purity”.

e Values determined by dividing the amount of HP-NAP from each purification step by that from the whole cell lysate.

### Structural and Molecular Properties of Recombinant HP-NAP Purified from *B. subtilis*

**Figure 6:**
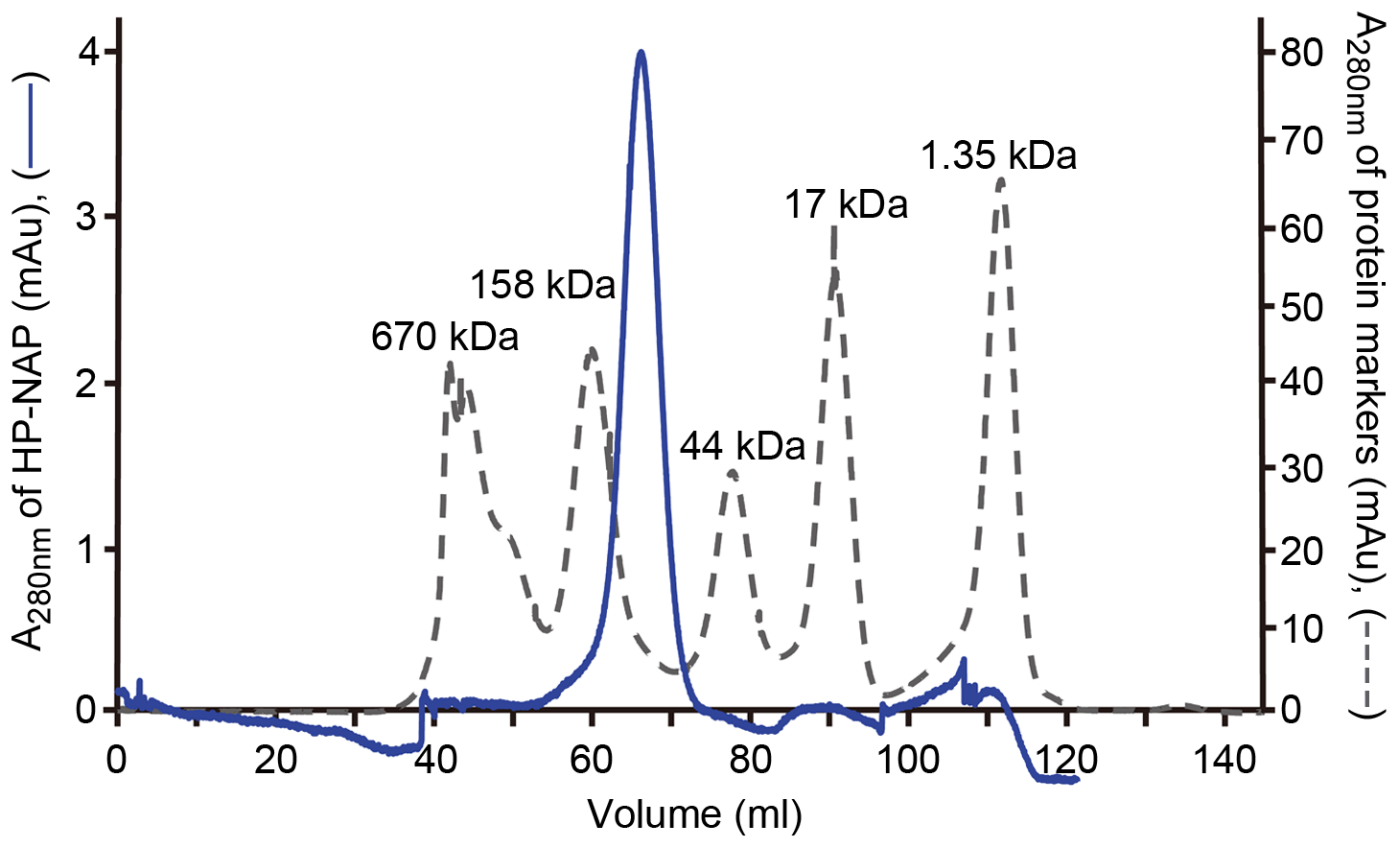
Gel-filtration chromatographic analysis of recombinant HP-NAP purified from *B. subtilis*. Purified HP-NAP and protein molecular weight makers were subjected to gel-filtration chromatography as described in Materials and Methods. The chromatograms were recorded by UV absorbance at 280 nm. The molecular weight of each protein marker was indicated at the top of each peak shown in the chromatogram.

**Figure 7:**
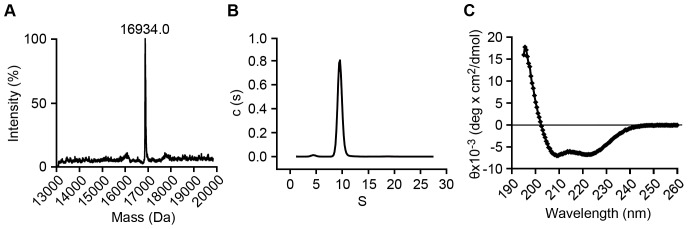
Analyses of molecular and structural properties of recombinant HP-NAP purified from *B. subtilis*. The molecular weight (**A**), sedimentation coefficient (**B**), and secondary structure (**C**) of HP-NAP were analyzed by liquid chromatography/electrospray ionization time-of-flight mass spectrometry (LC/ESI-TOF-MS), analytical ultracentrifugation, and circular dichroism spectroscopy, respectively, as described in Materials and Methods. A, The peak in mass spectrum is corresponding to the molecular weight of HP-NAP monomer. B, The sedimentation coefficient distribution c(S) is shown as a function of S. The c(S) distribution was analyzed using the software program SEDFIT. C, The far UV circular dichroism spectrum of HP-NAP was recorded at the wavelength range of 195 to 260 nm.

The structure and oligomeric state of the purified HP-NAP were examined to determine whether the recombinant HP-NAP purified from *B. subtilis* folded into its native structure. Gel-filtration chromatographic analysis showed that the purified HP-NAP was eluted as a major peak with a molecular weight of about 150 kDa (Fig. 6), which is consistent with the previous reports [5], [23]. However, this apparent molecular weight is much lower than the molecular weight of ∼232 kDa as determined by native-PAGE analysis (Fig. 5B) and the theoretical molecular weight of 203 kDa for the dodecameric HP-NAP (Table 2). In gel-filtration chromatography, the mobility of a protein depends on its size and shape. The low apparent molecular weight of HP-NAP determined by gel-filtration chromatography may be due to the reason that the overall shape of HP-NAP is more compact than those of the standard proteins used for calibration. As to native-PAGE, the protein net charge might have more influence on the mobility of a protein than its size and shape. Thus, the high apparent molecular weight of HP-NAP determined by native-PAGE may be due to a lesser negative net charge on the surface of HP-NAP. We then performed liquid chromatography-mass spectrometry (LC-MS) to determine the molecular weight of the monomer of the purified HP-NAP. The molecular weight of HP-NAP monomer was 16.934 kDa (Fig. 7A), indicating that the molecular weight of the dodecameric form of purified HP-NAP is ∼203 kDa. Sedimentation analysis further showed that the purified recombinant HP-NAP sedimented as a major peak at 9.53 S, by which the molecular weight was calculated to be approximately 200 kDa (Fig. 7B). These results suggested that the purified HP-NAP formed a dodecameric protein. The secondary structure of the purified HP-NAP was further examined by circular dichroism spectroscopy. The far UV trace showed a curve with characteristic of an α-helix (Fig. 7C). The structural and molecular properties of HP-NAP obtained in this study are similar to those shown in the previous reports (Table 2). Thus, the recombinant HP-NAP purified from *B. subtilis* was folded into its native form as a multimeric protein with a secondary structure of α-helix.

**Table 2:** Comparison of the molecular properties of HP-NAP characterized from this and other studies.

SDS-PAGE (kDa)^a^	Gel-filtration chromatography (kDa)^a^	Native-PAGE (kDa)^a^	LC-MS(kDa)^a^	Sedimentation coefficient (s)/molecular weight (kDa)^b^	Secondary structure	References
∼17	∼150	∼232	16.934	9.53/∼200	α-helix	This study
∼15	∼150	N/D^c^	N/D	N/D	N/D	[5]
∼17	∼150	∼232	N/D	9.38/N/D	α-helix	[23]
N/D	N/D	N/D	N/D	9.9/∼200	N/D	[24]
N/D	N/D	N/D	16.875	N/D	α-helix	[9]
N/D	N/D	N/D	N/D	N/D	α-helix	[8]

a The experimental molecular weights calculated from the indicated measurements.

b The molecular weight calculated from sedimentation coefficient.

c N/D: Not determined.

NOTE: The theoretical molecular weights of HP-NAP were 16.933 kDa and 203.196 kDa for its monomer and dodecamer, respectively. The molecular weight of HP-NAP monomer was predicted from ExPASy (http://expasy.org/).

### Production of ROS in Human Neutrophils Induced by Recombinant HP-NAP Purified from *B. subtilis*

**Figure 8:**
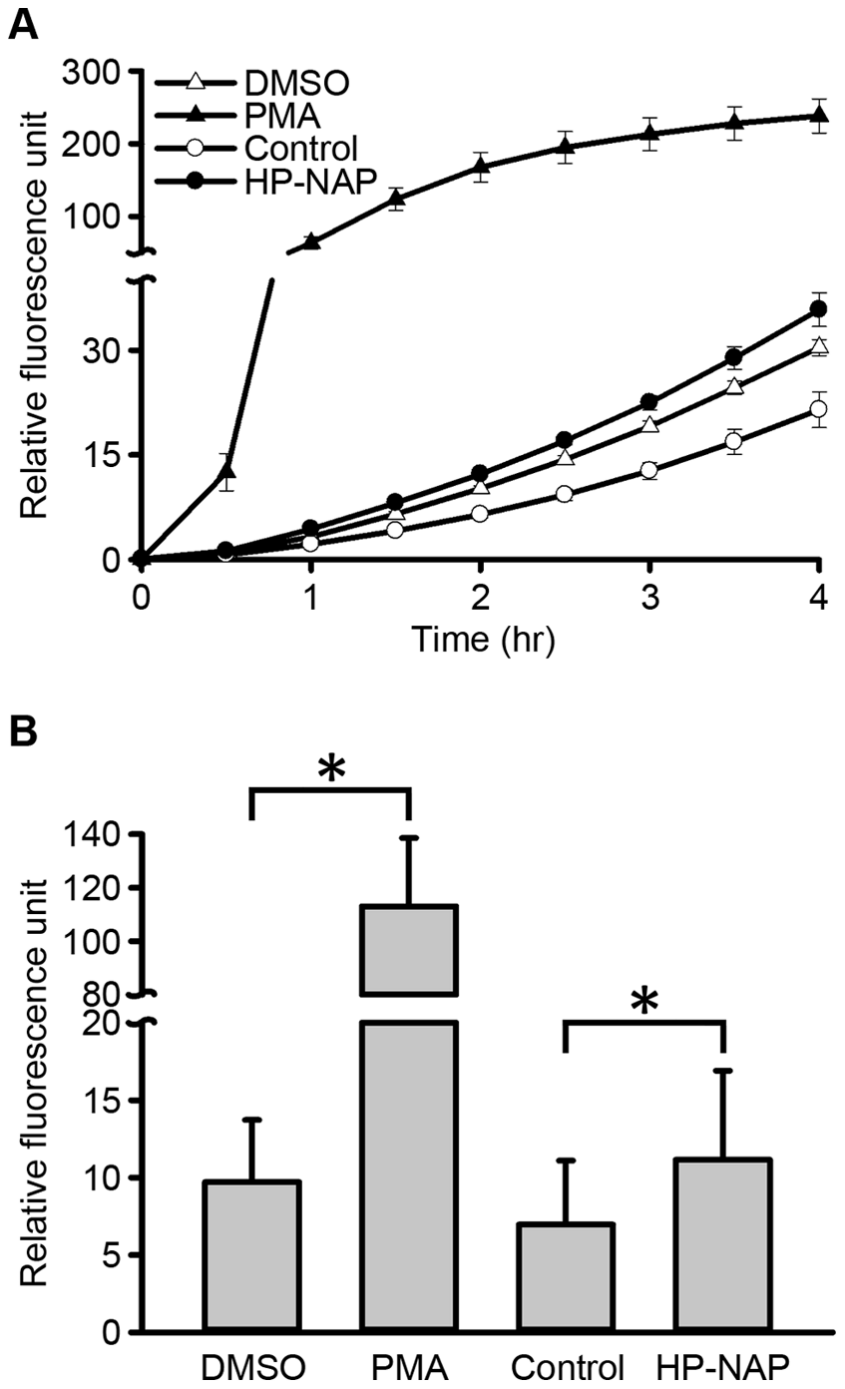
Production of reactive oxygen species in human neutophils induced by recombinant HP-NAP purified form *B. subtilis*. A, Human neutrophils (1×10^5^ cells) were treated with 1 µM HP-NAP, 0.08 µM phorbol 12-myristate 13-acetate (PMA) as a positive control, D-PBS and 0.05% DMSO in D-PBS as negative controls at 37°C for 4 hr. The contents of ROS generated in neutrophils were measured continuously by using a 2′, 7′-dichlorodihydrofluorescein diacetate (H_2_DCF-DA)-dependent assay as described in Materials and Methods. The result was represented as the profile of one experiment in triplicate. B, The fluorescent intensities detected from human neutrophils treated with indicated stimuli for 1.5 hr as described in (**A**) are shown. Data were represented as the mean ± S.D. of six independent experiments in triplicate (*p<0.01).

The biological activity of recombinant HP-NAP purified from *B. subtilis* was determined by assessing the ability of HP-NAP to induce ROS production in human neutrophils using 2′,7′-dichlorodihydrofluorescein diacetate (H_2_DCF-DA), a redox-sensitive fluorescent dye. The amount of ROS production is represented by the fluorescence intensity. After HP-NAP stimulation, a continuous increase of fluorescence in neutrophils was observed (Fig. 8A). The kinetics of the fluorescence increase in HP-NAP-stimulated neutrophils were much faster than those in control cells (Fig. 8A). Stimulation of neutrophils with HP-NAP for 1.5 hr significantly increased the fluorescence intensity by 1.6-fold, indicating that HP-NAP was capable of inducing ROS production in human neutrophils (Fig. 8B). Thus, the recombinant HP-NAP purified from *B. subtilis* by this negative chromatography approach kept its biological activity in stimulating ROS production in human neutrophils.

## Discussion

In this study, we have developed a new strategy to allow one-step chromatographic purification of the recombinant HP-NAP expressed in *B. subtilis*. The strategy was designed to obtain high purity of recombinant HP-NAP from flow-through using DEAE Sephadex anion-exchange chromatography. At pH 7.5 and 8.0, HP-NAP did not bind to the resin, whereas the majority of the other proteins from *B. subtilis* did bind to the resin. Under the optimized condition at pH 8.0, recombinant HP-NAP can be purified from *B. subtilis* by negative chromatography with DEAE Sephadex resin in one step. The recombinant HP-NAP purified from *B. subtilis* by using this negative chromatography approach was a multimeric protein with a secondary structure of α-helix and was able to stimulate human neutrophils to produce reactive oxygen species. Since recombinant HP-NAP was obtained in high purity using DEAE Sephadex anion-exchange chromatography in flow-through mode, this one-step negative chromatography was proved to be an efficient method for purifying the recombinant HP-NAP expressed in *B. subtilis*.

The finding that HP-NAP binds to the anion-exchange resin at pH 7.0 but not at pH 8.0 is unexpected, since the net negative charge of HP-NAP should be higher at pH 8.0 than at pH 7.0. However, protein binding to charged surfaces is affected by many factors other than protein net charge, such as protein charge distribution, protein-surface orientation, surface charge density, counterion, and ionic capacity of the mobile phase [25], [26]. Even within a spherical protein like HP-NAP, the distribution of charged residues on its surface is not uniform. Besides, there are a large number of positively charged residues present on the surface of HP-NAP [8]. The reason for HP-NAP not binding to the DEAE anion-exchange resin at pH 8.0 might be the existence of electrostatic repulsion between HP-NAP and the resin caused by the positive charges present on their surfaces. If this is true, how can HP-NAP bind to the DEAE resin at pH 7.0? During chromatographic separations, the target protein can interact with other molecules present in the sample. It has been reported that the elution characteristics of target proteins could be altered by the presence of other proteins strongly adsorbed on the resin [27]. Therefore, it is possible that at pH 7.0, HP-NAP binds to the DEAE resin through the indirect interactions of other impure proteins in the crude cell extract. We have found that at pH 7.0, purified HP-NAP was still present in the flow-through (unpublished observation, Yang and Fu). This finding indicates that the positive charge on the protein surface could be a dominant factor for HP-NAP not binding to the DEAE resin at pH 7.5 and 8.0.

So far, several methods for purification of recombinant HP-NAP have been reported. In our previous study, gel-filtration chromatography was performed twice to purify recombinant HP-NAP expressed in *E. coli* [23]. In some other studies, ion-exchange chromatography was applied to purify recombinant HP-NAP, but a secondary purification step utilizing gel-filtration chromatography to obtain highly pure HP-NAP was necessary [21], [22], [23]. In the present study, recombinant HP-NAP with high purity can be obtained in one step by using DEAE Sephadex chromatography in negative mode. Although one-step purification of HP-NAP has been achieved by affinity chromatography of the recombinant HP-NAP with a fusion peptide or protein [18], [28], [29], an extra step to remove the fusion peptide or protein from the purified recombinant HP-NAP might need to be considered to meet the requirement of its application. This extra step also complicates the purification procedure. Therefore, our approach using negative chromatography with DEAE Sephadex resin offers a simple and efficient method to purify HP-HAP in its native form.

There are few studies using *B. subtilis* as an expression system for HP-NAP production to reduce lipopolysaccharide contamination [9], [30]. In one of these studies, recombinant HP-NAP was purified as a soluble protein with purity higher than 50% by salting out of the endogenous proteins from *B. subtilis* with ammonium sulfate [30]. However, an extra step using metal chelate chromatography by the nickel chelating Sepharose FF column was needed to achieve higher purity [30]. The method applied herein was one-step DEAE Sephadex anion-exchange chromatography by collecting flow-through at pH 8.0 to obtain HP-NAP, of which the purity was higher than 94% (Table 1). The desalination step was not necessary because of low salt concentration in the flow-through, but the concentration step was needed to increase the protein concentration. After the purified HP-NAP was concentrated by using the ultrafiltration membrane with pore size of 30 kDa, its purity was further increased (Fig. 5). Thus, it is possible that an even higher purity of HP-NAP can be achieved by using a membrane with pore size higher than 100 kDa in the concentration step.

In conclusion, one-step negative chromatography using DEAE Sephadex resin has been developed to purify HP-NAP from *B. subtilis*. More than 99% of the endogenous proteins from *B. subtilis* were efficiently removed by DEAE Sephadex resin at pH 8.0, and the purity of HP-NAP was increased to at least 91%. The recovery of HP-NAP was higher than 60%. The purified HP-NAP was in its native form with biological activity. We expected that if a higher level of recombinant HP-NAP was expressed in *B. subtilis*, then a higher recovery and higher purity of recombinant HP-NAP could be achieved by using this purification method. The recombinant HP-NAP purified by this one-step chromatographic method could be further utilized for the development of new drugs, vaccines and diagnostics for *H. pylori* infection or for other new therapeutic applications.

## Materials and Methods

### Ethics Statement

Human blood was collected from five healthy volunteers with prior written informed consent and approval from the Institutional Review Board of the National Tsing Hua University, Hsinchu, Taiwan.

### Isolation of Human Neutrophils

A volume of 10 ml peripheral venous blood was drawn in vacuum blood collection tubes with sodium heparin. Neutrophils were isolated essentially as previously described [31]. Briefly, heparinized blood was mixed with an equal volume of 6% dextran in phosphate buffered saline (PBS), pH 7.4, containing 1.06 mM KH_2_PO_4_, 155.17 mM NaCl, and 29.66 mM Na_2_HPO_4_ (Invitrogen, Carlsbad, CA, USA) and incubated at room temperature for 45 min to sediment erythrocytes. After dextran sedimentation, the leukocyte-rich plasma was centrifuged at 340×*g* at 4°C for 10 min. The leukocyte pellet was resuspended in 3 ml ice-cold PBS, pH 7.4. The remaining erythrocytes were lysed by the addition of 3 ml red blood cell lysis buffer containing 0.15 M NH_4_Cl, 0.01 M KHCO_3_, and 5% EDTA, pH 7.2–7.4, at room temperature for 3 min. The above solution was then diluted to 50 ml by ice-cold PBS, pH 7.4, and subjected to centrifugation at 340× *g* at 4°C for 5 min. The leukocyte pellet was resuspended in 3 ml ice-cold PBS, pH 7.4, and the leukocyte suspension was layered onto Ficoll-Paque PLUS (GE Healthcare Life Sciences, Uppsala, Sweden) followed by centrifugation at 450× *g* at 4°C for 30 min. The cell pellet was then resuspended in 2 ml ice-cold Dulbecco’s phosphate buffered saline (D-PBS), pH 7.2, containing 1.5 mM KH_2_PO_4_, 137 mM NaCl, 8.1 mM Na_2_HPO_4_, and 2.7 mM KCl (Sigma-Aldrich, St. Louis, MO, USA) with the addition of 5 mM D-glucose (D-PBS-G) and kept on ice until needed. The final cell suspension, as judged by light microscopic examination at 400× magnification of at least 700 cells on Liu’s stained cytocentrifuged slides, contained >97% neutrophils with a viability exceeding 92% as gauged by the trypan blue exclusion test.

### Cloning of napA into *B. subtilis* Expression System

The *napA* gene was amplified by PCR from genomic DNA of *H. pylori* strain 26695 (Genbank Accession number AE000543) and then cloned into pCR4-TOPO vector using the TOPO TA cloning kit (Invitrogen, Carlsbad, CA, USA) as previously described [23]. The resulting plasmid, designated as pCR4-TOPO-NAP, was sequenced to confirm the correct insertion of *napA*. The DNA fragment containing *napA* was digested from pCR4-TOPO-NAP with *Nde*I and *Hind*III and then cloned into the pET28a expression vector (Novagen, Madison, WI, USA) to obtain the recombinant plasmid pET28a-His-NAP. The DNA fragment of *napA* was digested from pET28a-His-NAP with *Nde*I and *Xho*I and then cloned into the pRPA expression vector, a derivative vector of pEX5A [32]. The resulting plasmid was designated as pRPA-NAP. The map of pRPA-NAP is shown in figure S2.

The plasmid pRPA-NAP was transformed into a multiple-protease-deficient *B. subtilis* DB104 strain [33] by electroporation to express recombinant HP-NAP. The electro-transformation competent *B. subtilis* cells were prepared as previously described [34]. For electro-transformation, 100 µl of *B. subtilis* DB104 electro-transformation competent cells were gently mixed with 1 µl of pRPA-NAP (0.34 µg) and then transferred into a prechilled electroporation cuvette with 2 mm gap (Molecular BioProducts, San Diego, CA, USA). The cuvette was placed on ice for 5 min, and electroporation of pRPA-NAP into *B. subtilis* DB104 was carried out at field strength of 8.75 kV/cm, capacitance of 25 µF, and resistance of 500 Ω by a Gene Pulser Xcell™ Electroporation System (Bio-Rad, Hercules, CA, USA). The cells were then added in 1 ml of 2x Luria-Bertani (LB) recovery medium containing 3% tryptone, 1% yeast extract, and 1% NaCl with shaking at 120 rpm at 37°C for 2 hr. The transformed *B. subtilis* cells were selected by screening colonies on LB agar plates containing 10 µg/mL tetracycline.

### Expression of HP-NAP in B. subtilis

The selected *B. subtilis* DB104 colony containing pRPA-NAP (*B. subtilis* DB104- pRPA-NAP) was grown in LB medium supplemented with 10 µg/mL tetracycline at 37°C with rotary shaking at 150 rpm overnight. The overnight culture was then inoculated into 25 ml LB medium supplemented with 10 µg/mL tetracycline in a volume ratio of 1%, and the resulting cultures were grown at 37°C for 15 hr with rotary shaking at 180 rpm until the absorbance at 600 nm reached 2.0 to 2.1. For large-scale expression of recombinant HP-NAP in *B. subtilis*, the overnight culture of *B. subtilis* DB104-pRPA-NAP with absorbance of 2.0 to 2.1 at 600 nm was inoculated into 200 ml LB medium with supplemented 10 µg/ml tetracycline in a volume ratio of 1%. The resulting culture was then grown at 37°C with rotary shaking at 180 rpm until the absorbance at 600 nm reached 1.4 to 1.6. Afterwards, the cells were harvested by centrifugation at 6,000× *g* at 4°C for 15 min. The cell pellets were subsequently stored frozen at −70°C until purification.

### Lysis of *B. subtilis*

The *B. subtilis* cell pellets were lysed by either ultrasonication or by high pressure homogenization. For ultrasonication, the cell pellets were resuspended in an equal culture volume of 20 mM Tris-HCl, pH 8.0, and 50 mM NaCl with the addition of protease inhibitor mixture (PI mix) containing phenylmethylsulfonyl fluoride (PMSF), N-alpha-tosyl-L-lysinyl-chloromethylketone (TLCK), and N-tosyl-L-phenylalaninyl-chloromethylketone (TPCK) to a final concentration of 0.13, 0.03, and 0.03 mM, respectively, and the bacterial suspension was disrupted by an ultrasonic processor SONICS VCX-750 (Sonics & Materials, Newtown, CT, USA) on ice with 20% amplitude, independent ON and OFF pulse cycles of 1 sec, and processing time of 5 min. For high pressure homogenization, the cell pellets were resuspended in 1/10 culture volume of 20 mM Tris-HCl, pH 8.0, and 50 mM NaCl with the addition of PI mix, and the bacterial suspension was disrupted by a high pressure homogenizer (Avestin Inc., Ottawa, Canada) at 15,000 psi for 3 passes.

### Optimization of Recombinant HP-NAP Purification by Batch Method

The cell pellets of *B. subtilis* DB104-pRPA-NAP obtained from large-scale expression of recombinant HP-NAP were resuspended in 20 mM Tris-HCl, pH 8.0, and 50 mM NaCl with the addition of PI mix. The bacterial suspension was disrupted by high pressure homogenization, and the cell lysates were centrifuged at 30,000× *g* at 4°C for 1 hr. For the experiment to optimize the buffer pH values, the pH of the supernatant was either kept at 8.0 or adjusted to 7.5 and 7.0 by the addition of 0.5 N HCl in a volume ratio of 0.5% and 0.7%, respectively. The supernatants with pH values of 7.0, 7.5, and 8.0, were loaded onto DEAE Sepharose (Amersham Pharmacia Biotech, Uppsala, Sweden) and DEAE Sephadex A-25 (Sigma-Aldrich, St. Louis, MO, USA) resins, which were pre-equilibrated with the above Tris-buffer at the same pH as the cell lysate supernatants. For the experiment to optimize the amount of proteins loaded onto the resins, the concentration of the supernatant was adjusted to 0.3 mg/ml with the above Tris-buffer at pH 8.0, and different volumes of the adjusted supernatant was loaded onto DEAE Sephadex A-25 resins to keep the ratio of protein to resin ranging from 0.5 to 1.3 mg/ml. The above supernatant/resin slurries were shaken on a rotator at 4°C for 30 min to ensure complete protein adsorption to the resin. Then, the slurries were centrifuged at 10,000×*g* at 4°C for 30 sec, and the supernatants were collected as “unbound supernatants”. An equal resin volume of Tris-buffer at the same pH as the supernatants was added onto the resins for washing. The slurries were shaken on a rotator for 10 min and then were centrifuged as described above to collect the supernatants as “wash fractions”. After washing five times, an equal resin volume of elution buffer containing 20 mM Tris-HCl and 1 M NaCl at the same pH was added onto the resins to elute proteins adsorbed on the resins. The slurries were shaken on a rotator for 10 min and then were centrifuged as described above to collect the supernatants as “bound fractions”. This elution step was repeated twice. The fractions including unbound supernatant, wash fraction, and bound fraction were analyzed by SDS-PAGE and native-PAGE.

### Purification of Recombinant HP-NAP in *B. subtilis*

The cell pellets of *B. subtilis* DB104-pRPA-NAP obtained from large-scale expression of recombinant HP-NAP were resuspended in 20 mM Tris-HCl, pH 8.0, and 50 mM NaCl with the addition of PI mix. The bacterial suspension was disrupted by a high pressure homogenizer, and the cell lysate was centrifuged at 30,000×*g* at 4°C for 1 hr. The supernatant was then loaded onto the XK 26/20 column (GE Healthcare Life Sciences, Uppsala, Sweden) prepacked with DEAE Sephadex A-25 resins, which were pre-equilibrated with 20 mM Tris-HCl, pH 8.0, and 50 mM NaCl by using ÄKTA*prime* system (Amersham Pharmacia Biotech, Uppsala, Sweden). The flow rate was 1 ml/min, and the column temperature was set at 4°C. The flow-through was collected in 3-ml fractions. The column was then washed with two column volumes (CVs) of 20 mM Tris-HCl, pH 8.0, and 50 mM NaCl at a flow rate of 5 ml/min. The volume of each collected fraction was 5 ml for the first CV and 10 ml for the second CV of the wash step. Finally, the proteins adsorbed on DEAE Sephadex resins were eluted with 2 CVs of 20 mM Tris-HCl, pH 8.0, and 1 M NaCl at the same flow rate, and the eluate was collected in 10-ml fractions. The flow-through, wash, and elution fractions were analyzed by SDS-PAGE. The flow-through fractions containing HP-NAP were pooled and concentrated with a buffer exchange to D-PBS, pH 7.2, by using a stirred ultrafiltration cell (Amicon, model 8050) equipped with an Ultracel regenerated cellulose YM-30 membrane (Millipore, Billerica, MA, USA). The pooled fraction was added to the same volume of D-PBS, pH 7.2, and then concentrated back to its original volume. This procedure was repeated twice for buffer exchange. This protein solution was added to D-PBS, pH 7.2, in a volume ratio of 9 to 4 and concentrated to 7/10 of the initial sample volume. Then, the same procedure was repeated twice expect that the protein solution was concentrated to 1/2 of the initial sample volume for each concentration step. Finally, the protein solution was added to D-PBS, pH7.2, in a volume ratio of 3 to 2 and then concentrated to a final concentration of 0.3 mg/ml. During concentration, the buffer was exchanged to D-PBS, pH 7.2. The HP-NAP was then filtered through an Acrodisc with Mustang E membrane (Pall, Cortland, NY, USA) to eliminate the possible endotoxin contamination during the purification. The purified HP-NAP was stored at 4°C no more than one month. The amount of endotoxin was less than 25.35 endotoxin unit (EU)/mg of HP-NAP as determined by Super Laboratory Company (Taipei, Taiwan) using an enzyme-linked immunosorbent assay (ELISA) with the detection limit of 0.005 to 2 EU/ml. In addition to SDS-PAGE, purified recombinant HP-NAP was routinely analyzed by gel-filtration chromatography and native-PAGE to confirm its multimeric properties. The percentage purity of HP-NAP was calculated from the intensities of protein bands on SDS-PAGE as follows: purity (%) = (intensity of HP-NAP)/(intensity of HP-NAP + intensities of impurity)×100. The intensities of protein bands were quantified by densitometry analysis using multi gauge software V3.0 (Fujifilm, Tokyo, Japan).

### Immunoblotting

*B. subtilis* cell lysates and protein samples from each step of the purification process were denatured in sample buffer by heating at 95°C for 5 min. The proteins were separated on a 15% SDS-PAGE gel, and then transferred onto a polyvinylidene difluoride (PVDF) membrane. The membrane was blocked with 5% fat-free milk in TBST containing 50 mM Tris-Hcl, pH 7.4, 15 mM NaCl, and 0.1% Tween-20 at room temperature for 1 hr and then incubated with the hybridoma culture supernatant containing mouse monoclonal antibody MAb 16F4 against HP-NAP [28] at a dilution of 1∶100 in TBST containing 5% bovine serum albumin (BSA) at 4°C overnight. The membrane was washed three times with TBST containing 5% fat-free milk, incubated with the horseradish peroxidase-conjugated mouse secondary antibody at a dilution of 1∶5000 in TBST containing 5% fat-free milk at room temperature for 1 hr, and washed with TBST three times. Chemiluminescent detection was performed using enhanced chemiluminescence (ECL) Western blotting detection reagents (PerkinElmer, Waltham, MA, USA) by LAS-3000 (Fujifilm, Tokyo, Japan).

### Gel-filtration Chromatography

A volume of 500 µl of the purified recombinant HP-NAP at 0.3 mg/ml in D-PBS, pH 7.2, was applied onto a HiLoad 16/60 Superdex 200 prep grade column (Amersham Pharmacia Biotech, Uppsala, Sweden), which was pre-equilibrated with D-PBS, pH 7.2, by using ÄKTA FPLC (Amersham Pharmacia Biotech, Uppsala, Sweden) and analyzed as previously described [23]. A volume of 200 µl of standard proteins for gel filtration (Bio-Rad, Hercules, CA, USA) was applied to the same column to estimate the molecular weight of recombinant HP-NAP.

### Analytical Ultracentrifugation

The sedimentation velocity experiment was performed on a Beckman Coulter ProteomeLab™ XL-I analytical ultracentrifuge equipped with a UV absorbance optical detection system, using a four-hole An60 Ti analytical rotor and a 12-mm aluminum double-sector centerpiece. The sample and reference sectors were filled, respectively, with 392 µl of HP-NAP at a concentration of 0.3 mg/ml in D-PBS, pH 7.2, and 412 µl of D-PBS, pH 7.2. Centrifugation was carried out at 41,000 rpm and at a temperature of 20°C for 186 min. The UV absorbance data were collected at a wavelength of 280 nm in the radial increment of 0.003 cm with a single reading at each radius by time intervals of 3 min per scan. The sedimentation coefficient distribution, c(s), and the molecular weight of HP-NAP were calculated by the software SEDFIT.

### Liquid Chromatography Mass Spectrometry

The monomeric molecular weight of recombinant HP-NAP was analyzed by liquid chromatography mass spectrometry. The amount of 0.6 µg recombinant HP-NAP at a concentration of 0.3 mg/ml in D-PBS, pH 7.2, was injected into an Agilent 1100 series (Agilent Technologies, Santa Clara, CA, USA) capillary high performance liquid chromatography (HPLC) with a MST C4 0.075×10 mm column (Thermo scientific, Waltham, MA, USA). Desalting was achieved by a gradient system of two mobile phases with a flow rate of 0.028 ml/min. Mobile phase A was 5% acetonitrile/95% water (v/v) with 0.1% formic acid, and mobile phase B was 95% acetonitrile/5% water (v/v) with 0.1% formic acid. The gradient was as follows: the mobile phase B was increased from 5% to 40% over 40 min with a hold time of 50 min and then increased to 70% over 60 min. The desalted HP-NAP was then analyzed by micromass Q-TOF (Waters, Milford, MA, USA).

### Circular Dichroism Spectroscopy

Recombinant HP-NAP at a concentration of 0.3 mg/ml in D-PBS, pH 7.2, was subjected to circular dichroism (CD) analysis. The CD spectra were recorded on AVIV 62A DS spectrometer (AVIV Biomedical, Inc., Lakewood, NJ, USA) at 25°C using a 1 mm path-length cuvette. The CD data were obtained from the average value of two scans from 260 to 195 nm with 1 nm bandwidth at 0.5 nm intervals. A reference spectrum of D-PBS, pH 7.2, was also recorded. The CD spectrum of recombinant HP-NAP was calculated by subtracting the reference spectrum. The mean residue ellipticity (MRE) in the far UV was calculated with the formula: MRE = θ/(10×L×C×N), where the θ, L, C, and N are the measured signal in millidegrees, path-length of the cuvette, molar concentration of the protein, and number of peptide bonds, respectively.

### Oxidative Burst Assay

The production of reactive oxygen species was measured by a fluorometer-based microplate assay as previously described [35] except that 2′,7′-dichlorodihydrofluorescein diacetate (H_2_DCF-DA) was used. Isolated human neutrophils were resuspended at 2×10^6^ cells/ml in D-PBS, pH 7.2, containing 0.5 mM D-glucose (D-PBS-G). Aliquots of 50 µl of cell suspension were dispensed into individual wells of a 96-well black plate (Nunc, Rochester, NY, USA) with a flat bottom. Subsequently, 150 µl of the mixture containing 0.9 mM CaCl_2_, 0.5 mM MgCl_2_, 0.27 µM H_2_DCFH-DA, and individual stimulus in the D-PBS, pH 7.2, was added into each well to a final volume of 200 µl, and then the cells were incubated at 37°C. H_2_DCFH-DA and phorbol 12-myristate 13-acetate (PMA) were dissolved in methanol and DMSO at concentrations of 10 mM and 0.2 mM as the stock solutions, respectively. Both of them were diluted into D-PBS, pH 7.2, containing 0.9 mM CaCl_2_ and 0.5 mM MgCl_2_ immediately before use. The final concentrations of recombinant HP-NAP, PMA, and H_2_DCFH-DA were 1, 0.08, and 0.2 µM, respectively. The fluorescence emission at 538 nm was monitored in triplicate every 30 min for 4 hr by Fluoroskan Ascent fluorometer (Labsystems, Helsinki, Finland).

### Miscellaneous Methods

Protein concentrations were routinely analyzed by the Bradford method using a commercial dye preparation (Bio-Rad, Hercules, CA, USA), and BSA was used as a standard. SDS-PAGE and native-PAGE were performed in gels containing 15% and 10% acrylamide, respectively. The native-PAGE was performed similar to SDS-PAGE except that the protein samples were supplemented with non-denaturing sample buffer containing 62.5 mM Tris-HCl, pH 6.8, 10% glycerol, and 0.01% bromophenol blue and that electrophoresis was performed at 4°C using Tris-glycine system without SDS. The recombinant HP-NAP used as a positive control in PAGE analysis was purified from *E. coli* BL21(DE3) cells harboring pET42a-NAP using two consecutive gel-filtration chromatography as previously described [23].

### Statistical Analysis

All data are represented as the mean ± standard deviation (S.D.). Statistical analyses were performed by using Excel 2010 software (Microsoft). The statistical significance was determined by Student’s *t*-test. A probability (*p*) value of less than 0.05 was considered to represent statistical significance.

## Supporting Information

Figure S1 **Native-PAGE analysis of the purification process of recombinant HP-NAP expressed in** ***B. subtilis*** **with two DEAE resins at different pH values by a batch method.** The protein samples are the same as those described in figure 2. The soluble fraction from the whole cell lysate of *B. subtilis* DB104-pRPA-NAP, indicated as load, and the unbound supernatant, wash fraction, and elution fraction collected using DEAE Sephadex (A) and DEAE Sepharose (B) resins were analyzed by native-PAGE. Molecular weights (M) in kDa are indicated on the left of the stained gels.
(TIF)

Figure S2 **The scheme of plasmid pRPA-NAP.** The *nap*A gene was subcloned into the *Nde*I and *Xho*I restriction sites of the pRPA vector. This resulting plasmid was designated as pRPA-NAP.
(TIF)
