# Supplementary figures and images for: One-Step Chromatographic Purification of Helicobacter pylori Neutrophil-Activating Protein Expressed in Bacillus subtilis
Source: PLoS One. 2013 Apr 8;8(4):e60786. doi: 10.1371/journal.pone.0060786 (PMC3620106; doi:10.1371/journal.pone.0060786)

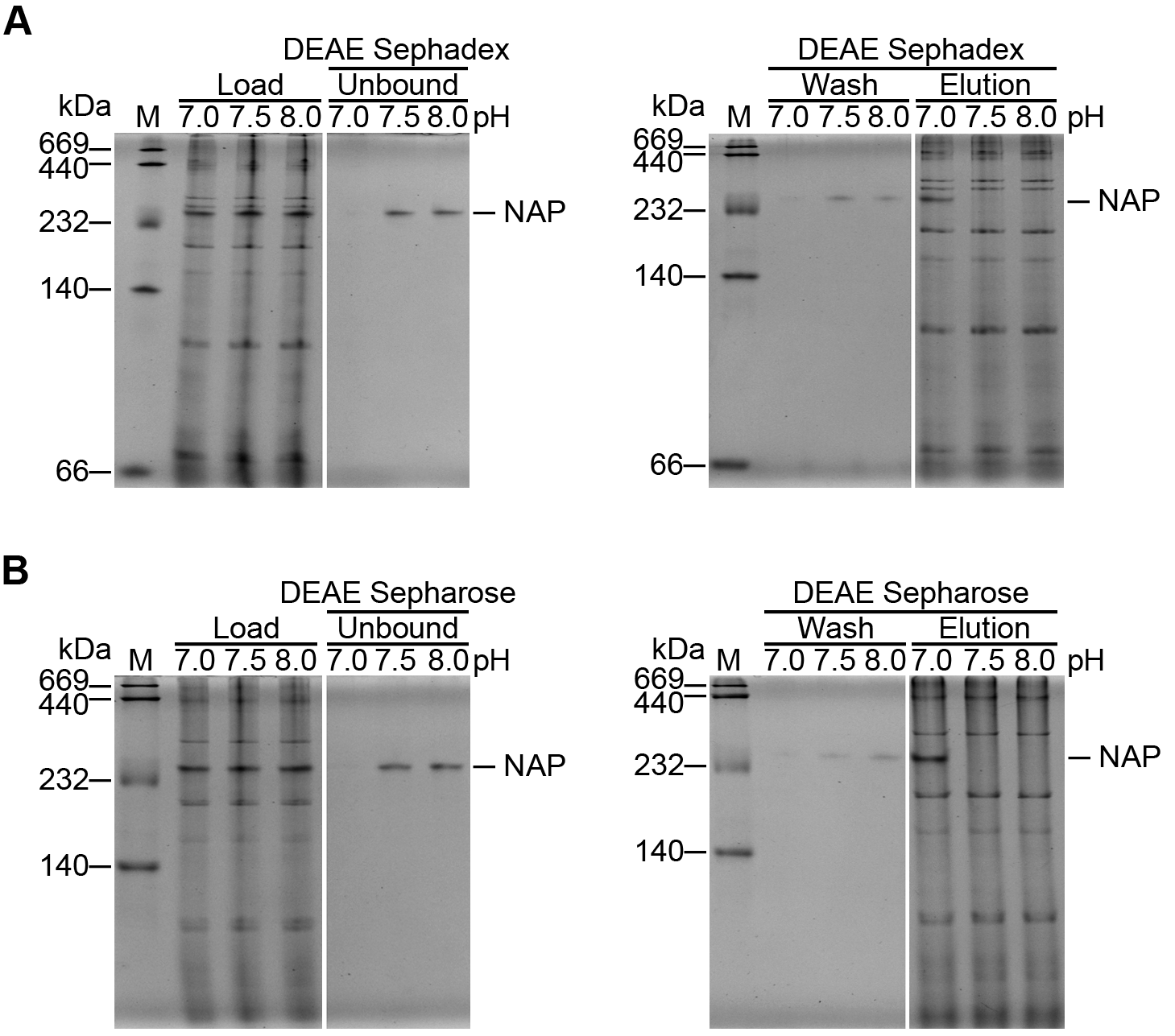

Supplement: Figure S1 — Native-PAGE analysis of the purification process of recombinant HP-NAP expressed in B. subtilis with two DEAE resins at different pH values by a batch method. The protein samples are the same as those described in figure 2. The soluble fraction from the whole cell lysate of B. subtilis DB104-pRPA-NAP, indicated as load, and the unbound supernatant, wash fraction, and elution fraction collected using DEAE Sephadex (A) and DEAE Sepharose (B) resins were analyzed by native-PAGE. Molecular weights (M) in kDa are indicated on the left of the stained gels. (TIF) [file pone.0060786.s001.tif]

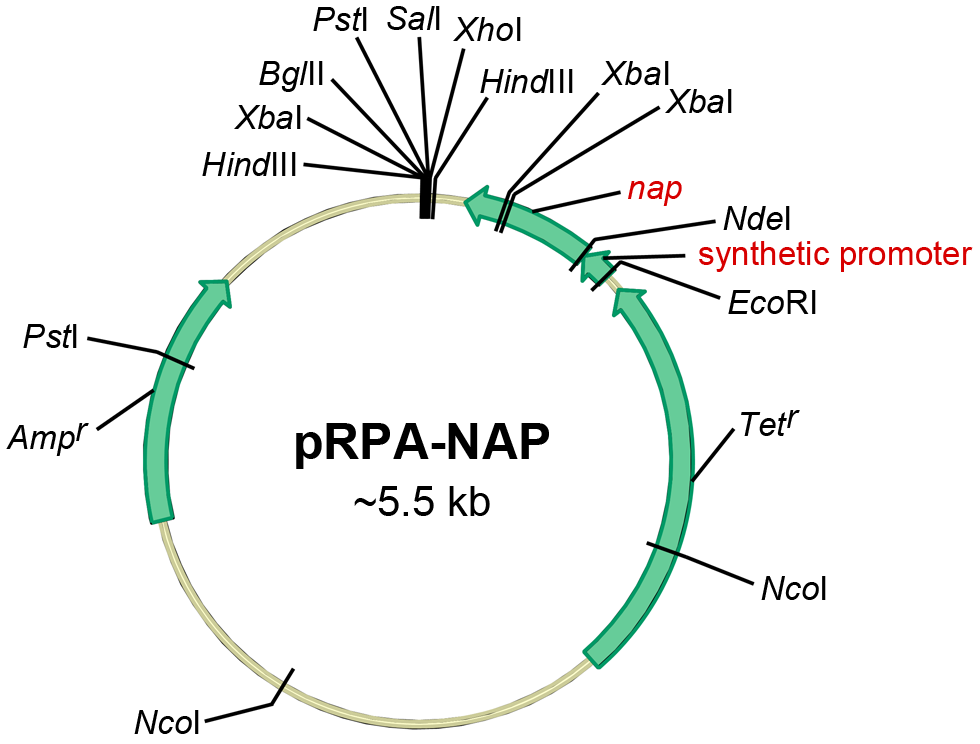

Supplement: Figure S2 — The scheme of plasmid pRPA-NAP. The napA gene was subcloned into the NdeI and XhoI restriction sites of the pRPA vector. This resulting plasmid was designated as pRPA-NAP. (TIF) [file pone.0060786.s002.tif]
